# Decadal Land-Use Change and Water Quality Degradation Reshape the Functional Structure of Fish Assemblages in Guangxi Rivers, China

**DOI:** 10.3390/ani16142253

**Published:** 2026-07-21

**Authors:** Huadong Yi, Leyao Lin, Sheng Bi, Han Lai, Dingli Guo, Xuchong Wei, Xuange Liu, Jiahui Chen, Shuang Liu, Chenlei Huang, Guifeng Li, Yong Zhang

**Affiliations:** 1 State Key Laboratory of Biocontrol, School of Life Sciences, Southern Marine Science and Engineering Guangdong Laboratory (Zhuhai), Guangdong Provincial Key Laboratory for Aquatic Economic Animals, Guangdong Provincial Engineering Technology Research Center for Healthy Breeding of Important Economic Fish, Sun Yat-Sen University, Guangzhou 510275, China; yihd3@mail2.sysu.edu.cn (H.Y.); linly53@mail2.sysu.edu.cn (L.L.); laih5@mail2.sysu.edu.cn (H.L.); weixuchong@163.com (X.W.); liuxg27@mail2.sysu.edu.cn (X.L.); chenjh563@mail2.sysu.edu.cn (J.C.); liush276@mail2.sysu.edu.cn (S.L.); huangchlei3@mail2.sysu.edu.cn (C.H.); 2 School of Life Sciences and Environmental Resources, Yichun University, Yichun 336000, China; bish3@mail.sysu.edu.cn; 3 College of Biological and Environmental Engineering, Guiyang University, Guiyang 550005, China; guodingli1987@126.com

**Keywords:** environmental factors, fish assemblages, functional diversity, river ecosystems, urbanization

## Abstract

Long-term changes in land-use and water quality degradation are key pressures on riverine ecosystems, yet their effects on the functional structure of fish assemblages remain insufficiently quantified. We conducted a decadal assessment of fish assemblages in four rivers experiencing rapid land-use transformation in Guangxi, China—the Zuojiang (ZY), Youjiang (YJ), Yujiang (YuJ), and Guijiang (GJ) rivers. Species-level functional traits describing body shape, vertical habitat position, feeding guild, trophic level, and maximum total length were used to calculate multiple functional diversity indices related to environmental variables using redundancy analysis and random forest models. Over the decade from 2013 to 2023, impervious surface and bare land increased significantly, while forest, shrubland, and grassland declined, coinciding with marked deterioration in water quality, including higher chlorophyll-a and lower dissolved oxygen concentrations (*p* < 0.05). Fish abundance and species richness decreased, and formerly dominant benthic cyprinids and loaches were replaced by tolerant and invasive taxa such as *Oreochromis niloticus* and *Tilapia zillii*. NMDS and PERMANOVA indicated a significant shift in community composition between 2013 and 2023 (R^2^ = 0.14, *p* < 0.001), with fish assemblages shifting toward small-bodied, pelagic, omnivorous, and laterally compressed species. Functional richness declined, whereas functional evenness and functional dispersion increased, indicating functional simplification and homogenization. Multivariate and machine learning models consistently identified increasing impervious cover and declining dissolved oxygen as the strongest predictors of these changes. Our results demonstrate that land-use intensification and water quality deterioration are associated with reduced functional integrity in Guangxi rivers and highlight the value of trait-based indicators for monitoring and managing biodiversity in rapidly urbanizing catchments.

## Simple Summary

Freshwater fish assemblages are increasingly affected by land-use change and water quality degradation, but changes in their ecological functions are often less visible than changes in species numbers. This study showed that increases in impervious surface and bare land were accompanied by higher chlorophyll-a concentrations and lower dissolved oxygen, suggesting increased eutrophication and oxygen stress. Fish communities shifted from assemblages containing more benthic and specialized species toward communities dominated by smaller, more tolerant, omnivorous, and pelagic species. Functional richness declined, indicating that these fish communities occupied a narrower range of ecological functions, whereas functional evenness and dispersion increased, suggesting functional reorganization and simplification. These findings highlight that species numbers alone may not fully reflect ecological degradation. Protecting riparian habitats, reducing nutrient inputs, and incorporating fish functional traits into biomonitoring programs may help maintain the ecological integrity of subtropical river ecosystems under ongoing human disturbance.

## 1. Introduction

Freshwater fish communities are highly sensitive to catchment alterations, particularly land-cover change and water quality degradation. Urbanization and deforestation can increase impervious surfaces and remove riparian vegetation, altering stream hydrology and degrading habitat structure [1]. Concomitantly, nutrient loading and eutrophication (e.g., elevated chlorophyll-a) often lead to hypoxia and algal blooms, further stressing fishes [2]. These pressures typically reduce native species richness and favor tolerant, generalist, or invasive fishes, while altering the trophic structure [3]. Globally, land-use change is a major driver of freshwater biodiversity loss [4], often interacting with climate change to promote warm-water- and slow-flow-adapted species, a process known as community thermophilization.

Trait-based indicators have become an important complement to traditional taxonomic metrics in aquatic bioassessment. Attributes such as fish body shape, feeding guild, and life-history strategy are directly linked to ecological function and environmental tolerance [1]. Functional diversity indices (e.g., richness, evenness, dispersion) summarize the distribution of these traits across the community. Assessing multiple dimensions of biodiversity provides a more complete signal of ecological change; for example, functional richness (FRic), functional evenness (FEve), and functional dispersion (FDis) can reveal changes in the occupied trait space, such as the loss of specialized morphologies or the proliferation of generalist feeders, which are not apparent from species counts alone [5]. Recent studies have applied multivariate and machine-learning approaches (RDA, NMDS, PERMANOVA, random forests) to link fish trait metrics with environmental drivers, demonstrating their utility in predicting fish community responses to habitat change [6].

Despite China’s rapid urbanization, long-term trait-based studies of riverine fish assemblages remain scarce. The Guangxi Zhuang Autonomous Region is a global freshwater biodiversity hotspot, with more than 380 freshwater fish species recorded there [7]. Yet, many catchments in Guangxi are undergoing rapid land-use conversion and increasing pollution. A recent analysis in subtropical China (Shenzhen) found that strongly urbanized watersheds supported a significantly lower taxonomic and functional fish diversity and a higher proportion of non-native species than forested watersheds [3]. However, comparable decadal surveys using functional metrics have not been conducted in Guangxi’s rivers. Filling this gap is essential for regional conservation planning and ecosystem management. However, comprehensive trait-based analyses using decades of monitoring data are rare. In particular, how decadal trends in land use and water quality in mixed-use catchments influence fish functional diversity remains poorly understood. This knowledge gap limits our understanding of how anthropogenic changes affect the functional integrity and resilience of freshwater ecosystems over time. To understand the consequences of these changes, researchers increasingly apply a functional trait perspective. Functional diversity (FD) refers to the variety of biological traits in a community that influence ecosystem functioning [8]. For fishes, relevant traits include body size, diet, habitat preference, and tolerance to pollution, which together determine their roles in food webs and nutrient cycles. Functional diversity can be quantified using indices such as functional richness, which measures the volume of trait space occupied by a community, functional evenness, which reflects how evenly species abundances fill that space, and functional dispersion or divergence, which describes how far species traits lie from the community centroid [9].

To address the gap in research, we analyzed decadal changes in fish assemblages from 2013 to 2023 across four major rivers in Guangxi: Zuojiang River (ZY), Youjiang River (YJ), Yujiang River (YuJ), and Guijiang River (GJ). We quantified changes in trait composition, including body shape, trophic level, and reproductive guild, and calculated community-level functional richness, evenness, and dispersion. We used redundancy analysis, NMDS, and PERMANOVA to evaluate overall community dynamics and applied random forest models to identify the watershed variables that most strongly explained these patterns, notably impervious cover, forest loss, chlorophyll-a, and dissolved oxygen. This multi-metric trait-based framework is well aligned with the aims of ecological indicators and can advance understanding of how anthropogenic pressures reshape riverine fish assemblages. Our findings provide practical guidance for the management and restoration of urbanizing catchments by clarifying key pressure–response relationships.

## 2. Material and Methods

### 2.1. Study Area and Sampling Design

**Figure 1:**
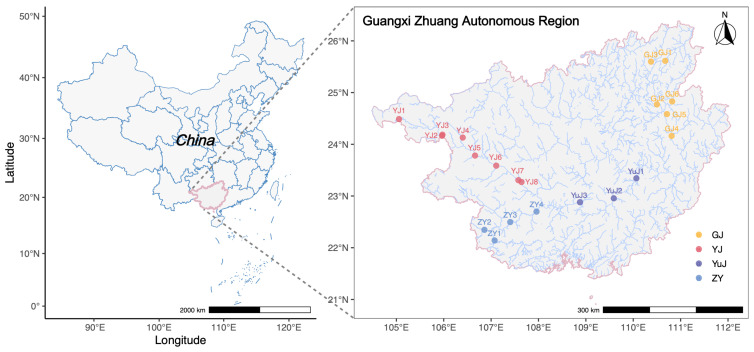
Map of the study area and distribution of sampling sites.

We analyzed two datasets (2013 and 2023) from 21 fixed sampling sites distributed along four river sections, namely, Zuojiang River (ZY), Youjiang River (YJ), Yujiang River (YuJ), and Guijiang River (GJ) (Figure 1). The geographic coordinates (longitude and latitude) of each sampling site are presented in Table S1. The 2013 fish and water quality data were obtained from a historical survey of major rivers in Guangxi Province, China, and no new animal sampling was conducted for the 2013 dataset in the present study. The fish and water quality data from the historical survey were collected in late March 2013. In 2023, we resurveyed the same sites in early April, corresponding to the same spring season and thereby improving the seasonal comparability between the two survey periods. During the 2023 survey, fishes were collected using standardized sampling gear consisting of multi-mesh gill nets and cage traps. Each multi-mesh gill net was 30 m long and 2.0 m high and consisted of 12 panels, each measuring 2.5 m × 2.0 m, with mesh sizes of 5, 6.25, 8, 10, 12.5, 15.5, 19.5, 24, 30, 35, 43, and 55 mm. Four floating gill nets, four bottom gill nets, and four cage traps were set per site to sample fish assemblages. In addition, cage traps were used to capture benthic fishes; each trap was 15 m long, 0.4 m wide, and 0.3 m high, with a mesh size of 5 mm and 20 entrances on each side. All nets and traps were deployed overnight for approximately 12 h and retrieved following a consistent procedure. In situ water quality parameters were measured at each sampling site. The 2023 fish survey was conducted as routine ecological monitoring of wild fish assemblages rather than as a laboratory experiment, captive animal trial, or invasive physiological manipulation study. All captured fishes were identified to the species level based on external morphological diagnostic characters, mainly following the Freshwater Fishes of Guangxi and Survey and Research on Fish Resources in Major Rivers of Guangxi in the Pearl River Basin [10,11]. Species names and current taxonomic status were further verified using FishBase, version 02/2026 [12], and abundance was recorded for each species. Fish handling time was minimized during field sampling, and live individuals were released whenever possible after identification. Specimens that could not be reliably identified in the field were retained only when necessary for taxonomic confirmation.

Five functional traits were assigned to each fish species: body shape, habitat preference (demersal/pelagic), feeding mode (e.g., benthic, omnivore, pelagic), trophic level, and maximum total length. These traits were selected to represent key ecological dimensions of fish assemblages, including morphology, habitat use, trophic strategy, and body size, which are widely used in trait-based analyses of fish communities. Body shape and maximum total length are associated with habitat use, swimming ability, and life-history strategy, while vertical habitat position, feeding mode, and trophic level reflect resource use and trophic function. Therefore, changes in these traits can indicate functional reorganization and potential changes in ecosystem functioning [9,13,14].

Trait assignment followed a standardized rule: categorical traits were assigned according to the dominant adult morphology, main adult habitat position, and primary feeding pathway reported in FishBase and supporting references, while trophic level and maximum total length were retained as continuous numerical variables [12]. When trait information was ambiguous, incomplete, or inconsistent across sources, the accepted scientific names or verified synonyms were checked in FishBase, and regional fish monographs were used for further verification. After this checking process, all species retained for the functional diversity analyses had complete trait records. Categorical traits were coded using a unified scheme, while trophic level and maximum total length were retained as continuous numerical variables. The detailed trait categories, coding scheme, and data sources are provided in Table S2.

Land-cover classifications for 2013 and 2023 were obtained from satellite imagery and GIS data [15], categorizing areas into forest, farmland, impervious surface (built-up), grassland, shrubland, bare soil, and water. At each sampling site, we calculated the percent cover of each land-use category within the local catchment. Key water quality variables (chlorophyll-a concentration, dissolved oxygen (DO), pH, electrical conductivity, and temperature) were measured using a Macro 900 multi-parameter water quality analyzer (Palintest Ltd., Gateshead, UK).

### 2.2. Data Analysis

All data analyses and visualizations were conducted in R (version 4.3.3). Figures were generated using the ggplot2 package (v3.5.1). One-way analysis of variance (ANOVA) was applied to assess differences across years. When significant effects were detected (*p* < 0.05), Tukey’s Honest Significant Difference (HSD) test was used for pairwise comparisons. The multcompView package (v0.1-10) was used to assign significance letters to different groups.

The alpha diversity of fish communities was quantified using the Shannon–Wiener index, Pielou’s evenness index, and species richness, calculated with the diversity(), evenness(), and estimateR() functions in the vegan package, respectively. The equations for these indices are provided in the Supplementary Materials. Beta diversity was calculated from species abundance data using the Bray–Curtis dissimilarity matrix, computed with the vegdist() function in vegan [16]. Non-metric multidimensional scaling (NMDS) was used to visualize community structure. To test the overall effects of grouping factors (e.g., year and watershed) on community structure, permutational multivariate analysis of variance (PERMANOVA) was conducted [17]. In addition, the betadisper() function was used to calculate and compare within-group dispersion (i.e., distances to group centroids). Functional diversity was assessed using the alpha.fd.multidim() function in the mFD package (v1.0.7).

To elucidate the driving effects of environmental factors on fish communities, several complementary analytical approaches were employed. First, Spearman rank correlations between environmental variables and functional diversity indices were calculated using the corr.test() function in the psych package, with the *p*-values adjusted using the false discovery rate (FDR) method. Second, Mantel tests were performed to assess the correlations between the Bray–Curtis distance matrix of fish communities and the Euclidean distance matrices of environmental factors (geographical, topographical, and water quality variables). In addition, β-diversity was partitioned into its turnover and nestedness-resultant components using the betapart package (v1.5.6) in R (version 4.3.3). Species abundance data were converted into a presence–absence matrix, and Jaccard dissimilarity was used to calculate total β-diversity and its turnover and nestedness components. The mean values obtained from the 2013–2023 pairwise comparisons were used to evaluate temporal patterns of species replacement and nested species loss/gain. Third, redundancy analysis (RDA) was performed to identify and quantify the key environmental factors explaining the spatial distribution of fish species composition. Finally, a random forest model was constructed using the randomForest package (v4.7-1.2), with NMDS axis scores as response variables and environmental factors as predictors. The relative importance of each environmental variable was evaluated and ranked based on the percentage increase in mean squared error (%IncMSE) [18]. Model performance was evaluated using five-fold cross-validation, out-of-bag (OOB) error analysis, and training–testing dataset comparison. The model validation metrics included the coefficient of determination (R^2^), root mean squared error (RMSE), and mean absolute error (MAE). Residual diagnostics were also performed to assess prediction bias. The detailed validation results are provided in Figure S1.

## 3. Results

### 3.1. Spatiotemporal Dynamics of Land-Use Change and Water Quality Factors

**Figure 2:**
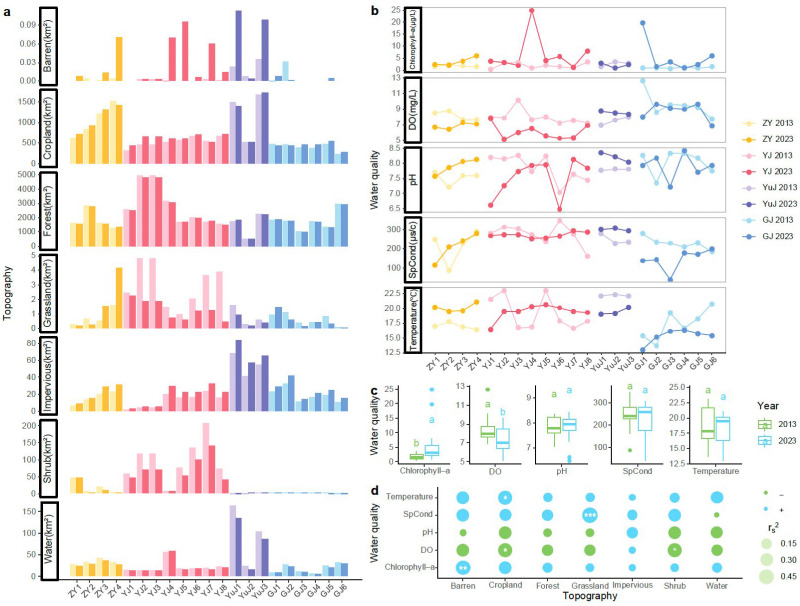
Spatiotemporal distribution characteristics and interrelationships of land-use composition and water quality factors at sampling sites. (**a**) Area proportions of different land-use types at each sampling site. (**b**) Distribution characteristics of water quality factors at each sampling site. (**c**) Significant differences in water quality factors between 2013 and 2023. Different lowercase letters indicate significant differences between years based on one-way ANOVA followed by Tukey’s HSD test (*p* < 0.05). (**d**) Heatmap of Spearman correlations between land-use area and water quality factors. Circle size indicates correlation strength based on the squared Spearman’s rank correlation coefficient (r_s_^2^), colors indicate the direction of the original Spearman correlation coefficient (r_s_), and asterisks indicate significance levels (* *p* < 0.05, ** *p* < 0.01, *** *p* < 0.001).

Between 2013 and 2023, watershed land cover changed markedly in the study area. Forest and cropland remained the dominant categories, while bare land, impervious surface, shrubland, and open water showed pronounced shifts. Overall, bare land and impervious surface areas increased across most sites, reflecting urban expansion, whereas shrub, water, and grassland areas generally declined (Figure 2a). Cropland area showed only modest increases in the Zuojiang and Youjiang River watersheds and little change in the Yujiang and Guijiang River watersheds. Forest cover tended to decrease overall (notably in YJ), and grassland expanded slightly in ZY but declined at other sites (especially in YJ). Impervious surface increased at all sites, with the largest increase in the Guijiang River basin, highlighting urban impact. Shrub cover declined at most sites (especially in YJ). Riverine water area remained mostly stable, though small reductions were observed in ZY and GJ.

Water quality variables also changed significantly (Figure 2b). In 2023, many ZY, YJ and GJ sites showed higher chlorophyll-a concentrations than in 2013, indicative of increased algal productivity. Dissolved oxygen concentrations declined markedly in ZY and YJ (with slight increases in YuJ). pH increased in ZY and YuJ but decreased in YJ. Conductivity rose in YuJ but fell in GJ. Temperature increased in ZY but decreased in YuJ and GJ. Notably, the 2023 chlorophyll-*a* concentration was significantly higher while the DO concentration was significantly lower than in 2013 (ANOVA, *p* < 0.05) (Figure 2c), indicating an overall trend toward eutrophication and hypoxia. Spearman correlations linked these trends to land cover: chlorophyll-*a* was positively correlated with bare land area, whereas DO was negatively correlated with cropland and shrub area; conductivity correlated positively with grassland, and temperature with cropland (Figure 2d). These patterns suggest that intensified agriculture and urbanization are associated with increased nutrient-related algal productivity and lower oxygen availability in the rivers.

### 3.2. Spatiotemporal Variation in Fish Abundance and Dominant Species

**Figure 3:**
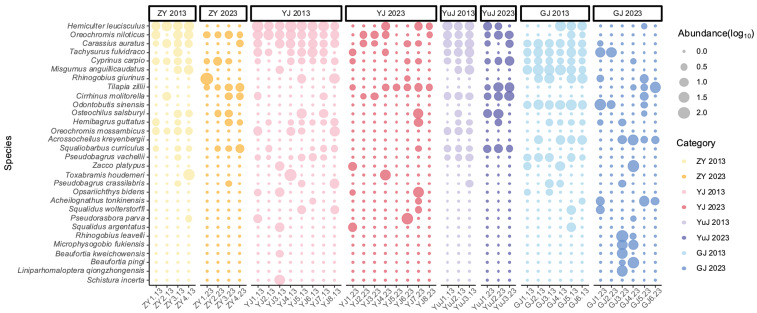
Bubble plot showing the spatiotemporal distribution of the top 30 fish species by abundance.

The fish community structure changed dramatically from 2013 to 2023. In 2013, the ZY, YJ, and YuJ rivers had very similar species compositions, dominated by tolerant benthic feeders such as *Hemiculter leucisculus*, *Oreochromis niloticus*, and *Carassius auratus*. The GJ river was internally similar, dominated by loach (*Misgurnus anguillicaudatus*), Chinese sleeper (*Odontobutis sinensis*), and yellow catfish (*Tachysurus fulvidraco*). In 2023, all four rivers showed clear compositional shifts (Figure 3). The total abundance of formerly dominant native species declined across the region. In the ZY, YJ, and YuJ rivers, the Nile tilapia (*O. niloticus*) and *Tilapia zillii* became the new dominant species, both of which are broadly tolerant, omnivorous species. In the GJ river, the sucker goby (*Rhinogobius leavelli*) had emerged as the dominant species by 2023. In short, the fish communities at these sites transformed from being dominated by locally native benthic feeders to being dominated by fast-growing, tolerant, and often introduced or generalist species.

### 3.3. Shifts in Fish Community Composition and Diversity Between 2013 and 2023

**Figure 4:**
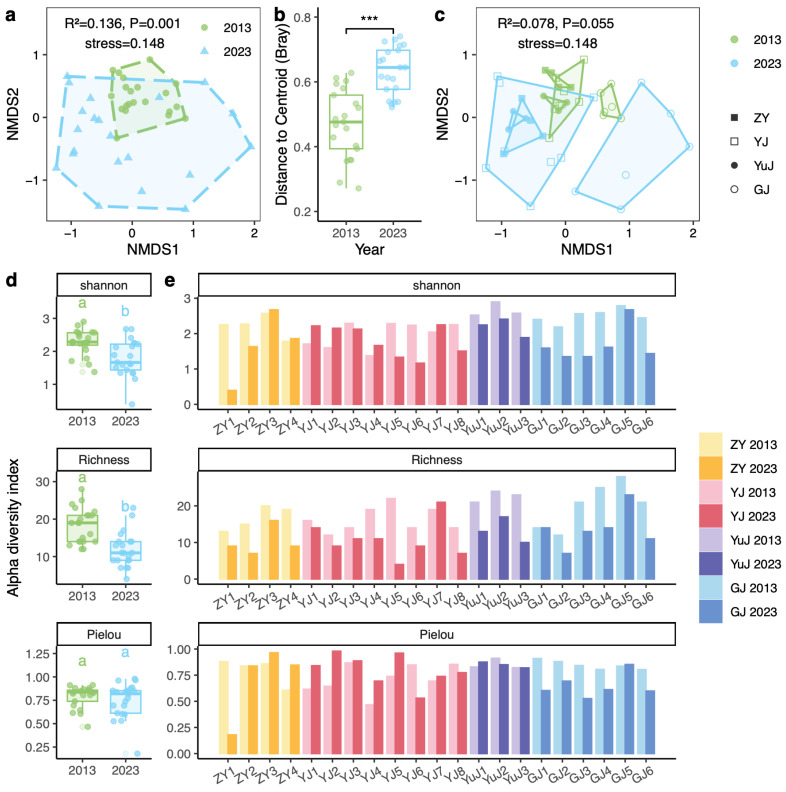
Spatiotemporal distribution characteristics of fish community composition. (**a**,**c**) Non-metric multidimensional scaling analysis of fish community based on Bray–Curtis distance. The analysis was grouped by year (**a**) and interaction between year and watershed (**c**), using multivariate analysis of variance with Adonis replacement, *p* < 0.001. (**b**) Bray–Curtis distance of fish community at the river sampling points between the two years. The asterisk indicates the level of significant difference between classifications with Kruskal–Wallis test (*** *p* < 0.001). (**d**) Analysis of fish α-diversity across the two years. Different lowercase letters indicate significant differences between years (one-way ANOVA with Tukey’s HSD multiple range test, *p* < 0.05). (**e**) Distribution characteristics of α-diversity of fish community.

Multivariate analyses revealed pronounced temporal shifts in overall community structure. The NMDS ordination showed a clear separation between samples from 2013 and 2023, with PERMANOVA confirming a highly significant year effect (Adonis R^2^ = 0.136, *p* < 0.001; Figure 4a). In addition, inter-site dispersion was substantially greater in 2023 than in 2013 (Figure 4b), indicating increased β-diversity and community heterogeneity. The interaction between year and river was weakly significant: the ZY, YJ, and YuJ fish communities remained relatively similar to one another, whereas the GJ fish community diverged markedly from the others in both years (Figure 4c).

Local α-diversity also declined over time (Figure 4d,e). Two-way ANOVA detected significant year effects for all indices, with species richness and Shannon diversity being significantly lower in 2023 than in 2013. Although YuJ and GJ maintained higher diversity than ZY and YJ, all rivers experienced declines, with the greatest reduction in Shannon diversity occurring in GJ. Species richness decreased significantly in all rivers, whereas Pielou’s evenness index showed no overall year effect except for a pronounced decline in GJ. Collectively, these results indicate that the fish communities in the four rivers lost species and became more uneven over the decade, reflecting reduced diversity and potentially lower ecosystem stability.

The β-diversity partitioning analysis further showed that the temporal community dissimilarity between 2013 and 2023 was mainly associated with species turnover rather than nestedness. The mean total β-diversity was 0.883, with the turnover component accounting for 93.57% of total β-diversity, while the nestedness-resultant component accounted for only 6.43% (Figure S2). This indicates that the decadal change in fish community composition primarily reflects species replacement rather than simple species loss or gain.

### 3.4. Spatiotemporal Variation in Fish Functional Traits and Functional Diversity

**Figure 5:**
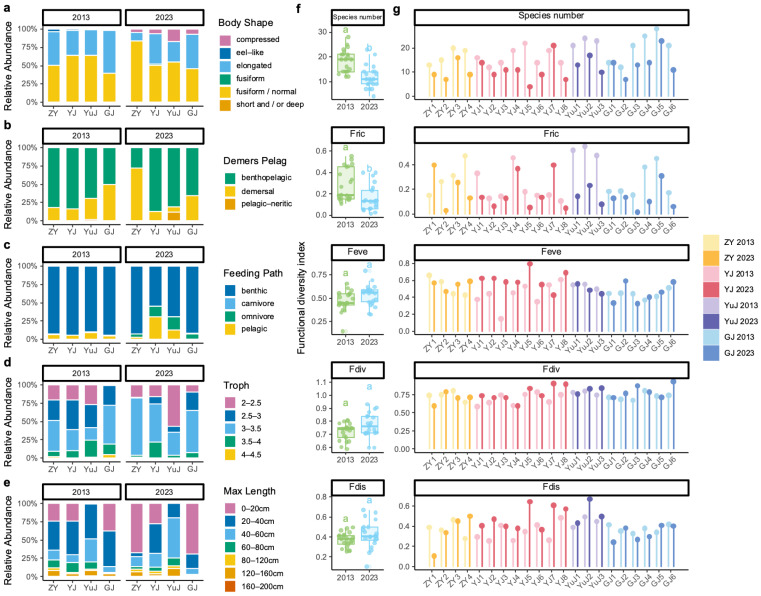
Spatiotemporal distribution characteristics of fish functional trait composition and functional diversity. (**a**–**e**) The relative abundance of fish functional traits (body shape, living water layer, diet, trophic level, and maximum body length) between the two years. (**f**) Analysis of functional diversity index across the two years. Different lowercase letters indicate significant differences between the years (one-way ANOVA with Tukey’s HSD multiple range test, *p* < 0.05). (**g**) The distribution pattern of functional diversity index.

The fish functional traits shifted markedly from 2013 to 2023 (Figure 5). The functional traits examined included body shape, vertical habitat position (Demers = demersal; Pelag = pelagic), feeding mode, trophic level (Troph), and maximum total length (MaxLength TL). The results for fish functional traits in 2013 and 2023 are provided in Supplementary Tables S2 and S3, respectively. In 2013, the fish communities in all rivers were dominated by species with fusiform (normal) and elongated body shapes; by 2023, fishes with compressed (laterally flattened) body shapes had become more prevalent, especially in YuJ, while fishes with elongated body shapes declined in ZY. Habitat preference shifted: benthopelagic (bottom-oriented) fishes were overwhelmingly dominant in 2013, but by 2023, their proportion had declined (notably in ZY) as the proportion of pelagic species increased. Feeding mode followed a similar pattern: benthic-feeding fishes were dominant in 2013 but had declined by 2023 across all rivers. Concurrently, omnivorous and pelagic-feeding species significantly increased, particularly in YJ and YuJ. The trophic-level structure changed as well: originally dominated by mid–low trophic levels (2.0–3.5), the fish communities in ZY, YuJ, and GJ exhibited a higher proportion of intermediate-trophic-level fishes (3.0–3.5) and relatively fewer high-trophic (>3.5) fishes in 2023. Finally, the body size trends showed that medium-sized fishes (40–80 cm) prevailed in 2013, while large (>80 cm) fishes were rare. By 2023, small-bodied fishes (0–20 cm) had become much more common (especially in ZY and GJ), and large fishes declined across all rivers.

These trait shifts were reflected in community functional diversity. Compared with 2013, all rivers had significantly fewer species and markedly lower functional richness (FRic) in 2023. This indicates a contraction of the fish community’s occupied functional trait space, indicating an effective loss of functional diversity. In contrast, functional evenness (FEve) increased, especially in YJ and GJ, suggesting that the remaining species were more evenly distributed within the reduced trait space. Functional dispersion (FDis) also rose in YJ, YuJ, and GJ, reflecting that trait values became more spread out (i.e., increased mean distance between species in trait space). Notably, functional specialization (functional uniqueness) increased in most rivers (especially in YJ and YuJ), indicating the emergence of a few species with extreme or unique trait combinations. Together, these changes imply that while the overall range of functions (FRic) has shrunk, the surviving species occupy the space more uniformly along with some novel trait outliers. In brief, from 2013 to 2023, the fish communities in the four rivers evolved from mostly benthic, normal-bodied, mid-trophic assemblages toward more pelagic/omnivorous, compressed-bodied, higher-trophic, and smaller-bodied assemblages, accompanied by lower functional richness and higher evenness, dispersion, and specialization.

### 3.5. Environmental Drivers of Fish Community Dynamics

**Figure 6:**
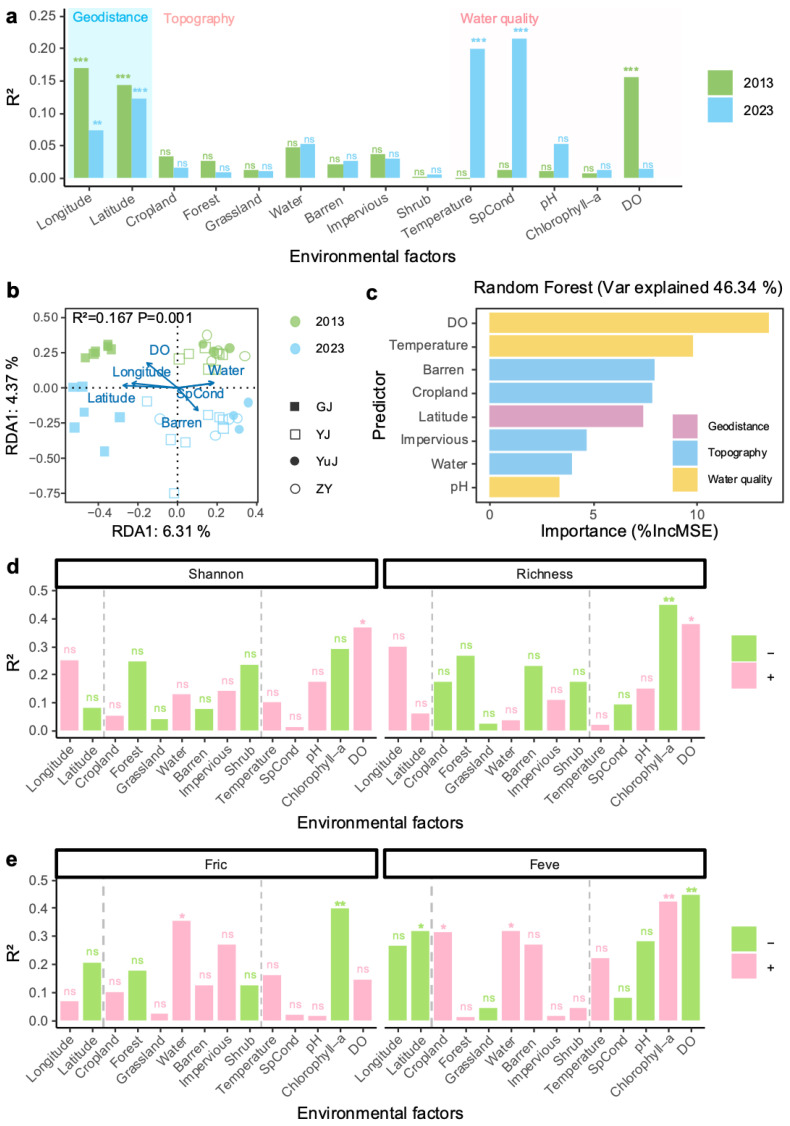
Multi-model analysis of environmental drivers of fish community assemblages. (**a**) Mantel test based on Bray–Curtis distance of fish community and three environmental variables (geographical distance, land-use type, water quality) in 2013 and 2023. (**b**,**c**) Key drivers of spatial distribution of fish communities identified using redundancy analysis (**b**) and random forest model (**c**). (**d**,**e**) Heatmap of Spearman correlations of environmental factors with alpha diversity (**d**) and functional diversity (**e**) of fish communities. Asterisks indicate significant correlation (* *p* < 0.05, ** *p* < 0.01, *** *p* < 0.001). “ns” indicates a non-significant correlation (*p* ≥ 0.05).

A suite of multivariate analyses highlighted shifts in the key drivers of fish spatial distribution. Mantel tests (Figure 6a) showed that in 2013, the fish community differences were primarily explained by geographic distance and dissolved oxygen concentrations. By 2023, the influence of geographic distance had weakened substantially, while water quality factors (notably temperature and conductivity) had become the dominant drivers of community composition. This suggests a shift from dispersal/historical constraints toward strong environmental filtering due to degraded water quality. The redundancy analysis (RDA) and random forest models corroborated these findings: both identified dissolved oxygen and temperature as the most important environmental predictors of community structure, while highlighting the significant effects of land-use variables (bare land, cropland, and impervious surface). In other words, factors tied to human activities and hydrological alteration (e.g., increased urban land cover), alongside declining DO and rising temperature, underlie the observed community turnover. Five-fold cross-validation of the random forest model indicated acceptable predictive performance and stability, yielding an average R^2^ of 0.6307 ± 0.1976, RMSE of 0.6410 ± 0.1918, and MAE of 0.5296 ± 0.1651. The OOB error stabilized after approximately 100 trees, and residuals were generally centered around zero, suggesting no obvious overfitting risk (Figure S1).

Spearman correlation analyses further clarified the relationships between environmental conditions and fish diversity. Chlorophyll-a, a proxy for eutrophication, was significantly and negatively associated with taxonomic and functional diversity, whereas dissolved oxygen showed positive correlations with these metrics. These patterns indicate that nutrient enrichment and the resulting oxygen depletion suppress fish diversity. Among habitat variables, water surface area was positively related to functional diversity, while cropland area showed a positive association with functional evenness. Notably, functional evenness increased with cropland, water area, and chlorophyll-a, but decreased with dissolved oxygen, suggesting that functional evenness rises as communities become increasingly dominated by generalist species under multiple stressors. Overall, the combined results of the multivariate and machine learning analyses demonstrate that declining water quality, driven by eutrophication and oxygen loss, and landscape change, stemming from urban and agricultural expansion, are the primary drivers of functional simplification and homogenization in these fish communities.

## 4. Discussion

### 4.1. Land-Use Change and Water Quality Impacts on Fish Assemblages

Urbanization and landscape disturbance alter hydrology and water chemistry, creating the classic “urban stream syndrome” of flashy flow, high nutrient/sediment loading, and tolerant biota [19]. In this study, the expansion of impervious surface intensified surface runoff and pollutant loading, which affected water quality and promoted algal blooms and chronic hypoxia. Over the past decade, accelerated urbanization and agricultural development have caused substantial water quality degradation and a concomitant simplification of the fish community structure in the ZY, YJ, YuJ, and GJ rivers. The pronounced increase in chlorophyll-a and the decline in dissolved oxygen concentrations indicate markedly elevated nutrient loading. Because the two surveys were conducted in comparable spring periods—in late March 2013 and early April 2023—seasonal variation is unlikely to fully explain the observed increase in chlorophyll-a. However, minor differences between the two sampling dates may still have contributed to the variation in chlorophyll-a observed, and this possibility should be considered when interpreting the results. The pattern of increased chlorophyll-a and decreased DO aligns with multiple studies showing that human expansion increases nutrient runoff, raising primary productivity (e.g., chlorophyll-a) and depleting DO [20]. For example, a previous study found that urban and agricultural land intensification elevates nutrient inputs and boosts algal biomass, while hypoxia from organic decomposition further lowers DO [20]. Similarly, another study [21] reported that in North America, urban runoff into streams raises nutrients and conductivity, warms the water, and reduces oxygen. Regarding the rivers examined in this study, forest and shrub cover declined while bare land and impervious surfaces rose (Figure 2a), which are the types of land-use changes expected to exacerbate runoff and nutrient flux. This concurrence of evidence confirms that accelerated land-use disturbance is the primary driver of the deteriorating water quality we detected. In short, our finding that change in the watershed’s land cover (due to urban/agricultural expansion) is significantly associated with increased chlorophyll-a and decreased DO is entirely consistent with the established links between land use and stream eutrophication [20].

Indeed, impervious-area models predict that runoff elevates sediments, nutrients, and contaminants while depressing dissolved oxygen (DO) [22]. We observed significant increases in chlorophyll-a and turbidity and decreases in DO in more urbanized reaches, which is consistent with degraded habitat quality. Sedimentation from agriculture or forestry likewise reduces spawning habitats and suffocates benthic feeders [22]. These mechanistic links explain why species richness and fish abundance plummet downstream: degraded water quality favors tolerant generalists. For example, when DO drops below the critical thresholds (often 5 mg/L), it eliminates sensitive species [23], allowing resilient invaders (e.g., tilapia) to proliferate. Overall, our results mirror the broader literature showing that land-cover change and pollution pose the greatest threats to river biodiversity [24]. Global assessments report that 25% of freshwater fishes are primarily threatened by pollution, construction of dams, agriculture, and invasive species [25], echoing our finding that nutrient enrichment and urban runoff have simplified the river environment. These drivers act in concert: cropland runoff and sewage effluent elevate nutrients (promoting algae and deoxygenation), while impervious surface drainage removes flow variability and habitat complexity [24]. Our structural equation and RDA analyses confirm that increased urban cover and low DO are the strongest predictors of fish community collapse, aligning with studies that highlight non-point pollution and low oxygen as key stressors of stream fishes [26].

Land use also interacts with local habitat changes in complex ways. Watershed alteration often degrades riparian zones and channel structure (e.g., straightening, bank loss), which affects stream temperature and flow regimes [27]. Riparian removal can warm stream water and increase sediments, directly impacting cold-water specialists [28]. Many studies have reported that changes in water chemistry (turbidity, conductivity, and nutrients) co-vary with land use, which together explain fish assemblage patterns. A previous study found that in tropical savanna headwater streams, only in-stream habitat and water quality variables (turbidity, dissolved oxygen, etc.) significantly influenced fish richness [29]. Likewise, a study conducted in northeastern China showed that fish taxonomic structure responded strongly to urban land-cover change and conductivity, while functional structure was tied to upstream forest and downstream agriculture proportions [27]. These findings imply that catchment urbanization (and its attendant runoff) tends to affect overall species composition, whereas agricultural and forested land cover may shape functional traits by modifying flows and refugia [27]. In any case, it is evident that land-use-driven degradation, often manifested as elevated nutrient and sediment loads and altered hydrological regimes, exerts a dominant influence on riverine fish assemblages [30,31]. Correspondingly, fish abundance and dominant species shift in ways that match these environmental changes. In our study, in 2023, the total fish abundance was found to be lower and the fish community was dominated by tolerant species in all rivers. The formerly dominant native taxa (e.g., *Hemiculter leucisculus*, *Carassius auratus*), which are relatively oxygen-demanding and specialist benthic feeders, had been largely supplanted by broad-diet, hypoxia-tolerant species (*Oreochromis niloticus* and *Tilapia zillii*) [32]. This pattern reflects species sorting under stress: generalist “weedy” species that thrive in eutrophic, low-oxygen conditions gain ecological advantage, while sensitive native species decline in number. The β-diversity partitioning results further support this interpretation, showing that the decadal compositional change was dominated by species turnover rather than nestedness, suggesting that the community reorganization mainly reflected species replacement rather than simple richness-driven species loss. Ecologically, this is expected: degraded environments favor fast-growing, generalist invaders or tolerant native species [33]. Human-disturbed streams in Brazil had lost many native specialists while pollution-tolerant species (often non-native) flourished [20]. In our case, the ascendance of *Oreochromis niloticus* and *Tilapia zillii* underlines the impact of warming and deoxygenation [34]. Even low-intensity disturbances can rapidly alter the community structure, and our results confirm that the moderate yet increasing human impact has been sufficient to drive pronounced species turnover in the four rivers. Similarly, studies in China and elsewhere have found that even modest logging or plantation conversion from primary forest leads to significant losses of fish species richness and functional trait richness [30]. The initial conversion of a Malaysian forest to oil palm plantation caused a sharp decline in taxonomic and functional richness, whereas further disturbance produced little additional effect. These results reinforce that land-cover disturbance and associated water quality decline are major drivers of fish diversity loss.

### 4.2. Trait-Based Evidence for Functional Simplification of Fish Assemblages Under Human Disturbance

In our study, water quality deterioration and habitat homogenization led to pronounced shifts in fish traits. Similar evidence has been reported in a previous study on Korean streams, where high nutrient and organic pollution levels were associated with significantly lower fish functional richness and fewer sensitive trophic groups, further supporting the close linkage between water quality degradation and fish functional diversity [35]. We observed a community-wide shift toward smaller, omnivorous, mid-water species along with the loss of large-bodied or benthic specialists, a pattern echoed in eutrophic or disturbed systems worldwide.

The fish diversity trends further illustrate these dynamics. All four rivers experienced significant declines in α-diversity indices over the decade. Species richness and Shannon diversity fell markedly, most steeply in the highly urbanized Guijiang river. Evenness showed a smaller net change, dropping significantly only in GJ. Such diversity loss indicates that many rarer or sensitive taxa were lost, causing community homogenization around a few tolerant species. The simultaneous increase in β-diversity (greater compositional differentiation between sites in 2023 than in 2013) suggests that the four rivers have lost species at different rates, likely due to spatially variable disturbance levels. In disturbed urban streams, diversity loss is often greater—supporting the “pollution–homogenization” hypothesis: heavily impacted areas become depauperate, while less disturbed areas retain more species, increasing between-site heterogeneity. This concurs with the patterns observed elsewhere; for example, Czeglédi et al. [32] reported strong diversity declines in highly urbanized Hungarian streams, and a similar phenomenon has been documented in North American urban wetlands. In our study, the greatest diversity loss observed in GJ correlates with its more extensive impervious-cover expansion, underscoring how urban intensity can amplify biodiversity loss. Together, the diminished local diversity and increased spatial heterogeneity imply that anthropogenic stress has unevenly filtered species across the river, which is consistent with global findings of human-driven biotic homogenization [34].

Water quality deterioration and habitat homogenization led to pronounced shifts in fish traits. We observed a community-wide shift toward smaller, omnivorous, mid-water species (e.g., laterally compressed cyprinids) along with the loss of large-bodied or benthic specialists, a pattern echoed in eutrophic or disturbed systems worldwide [1]. Eutrophication tends to favor planktivores and mobile predators while disadvantaging substrate-dependent species [36]. Consistent with our results, a previous study found that algal-rich Chinese lakes are dominated by pelagic planktivores with reduced trait diversity [36]. Functionally, our metrics capture this simplification: functional richness (FRic) declines as unique trait combinations disappear, while functional evenness and dispersion increase among the remaining generalists. In other words, selected tolerant fishes now evenly fill a narrower niche space. Functionally, our metrics indicate trait-space contraction and reorganization rather than simple functional homogenization. The decline in functional richness (FRic) suggests that the overall volume of the occupied trait space has decreased, likely reflecting the loss of or a reduction in specialist and intermediate trait combinations. In contrast, the increases in functional evenness (FEve) and functional dispersion (FDis) indicate that the remaining species are distributed more evenly within this reduced trait space and that some species occupy more divergent or outlying trait positions. Therefore, the combination of a lower FRic but higher FEve and FDis is best interpreted as trait-space compression with increased functional divergence among the remaining assemblages. Similar patterns have been reported in longitudinal studies, where FEve and FDis were observed to increase significantly after 30 years of urbanization in Texas streams, indicating functional reorganization under urban disturbance [1]. Likewise, species invasions in Chinese headwater streams have been associated with increased species richness but reduced functional richness, producing taxonomically rich but functionally simplified assemblages [37]. These trait changes imply that a part of the original functional trait space is lost, especially specialist or intermediate trait combinations, while tolerant generalists and some functionally distinct species persist or even increase. Our finding of increased FDis despite reduced FRic is, therefore, consistent with the broader evidence that degraded fish communities may undergo functional simplification together with trait redistribution, rather than uniform functional change alone [36].

The consequences of long-term changes in river fish diversity can be surprising. Many fish assemblages have not shown widespread taxonomic extinctions but rather species turnover. A recent global synthesis report highlights that fish species richness in many rivers has been stable or even slightly increasing over recent decades, often due to the introduction of non-native species [38]. Overall beta diversity (dissimilarity) has risen over time, with compositional turnover and nestedness increasing significantly each decade [38]. In other words, communities are being reshuffled even if total species counts do not decline. Similar patterns have been reported in Texas, where a 30-year study showed that the fish species richness of an urban stream remained stable while species composition turned over, with tolerant non-native species replacing sensitive native taxa [1]. In many cases, taxonomic alpha diversity is decoupled from beta diversity: high turnover and homogenization can accompany limited net loss in species numbers.

Functional diversity trends often diverge from taxonomic trends and may even precede them. In China’s Min River, functional richness (FRic) declined by more than 30% between 1979 and 2015, while functional nestedness increased, indicating that the fish communities became increasingly nested subsets [5]. Notably, a study found that functional composition responded to land-use change at lower disturbance thresholds than did taxonomic composition [28]; that is, shifts in species traits often emerge earlier, a pattern also observed in lakes where functional diversity is highly sensitive to eutrophication and fish stocking, even when taxonomic richness is less affected [39]. These findings suggest that functional metrics (dispersion and richness of trait space) can be more sensitive indicators of human impact.

The functional trait perspective reveals deeper community restructuring. We observed a clear shift from a community dominated by benthic, robust-bodied, mid-trophic-level fishes to a community dominated by pelagic and omnivorous, laterally compressed, higher-trophic-level, small-bodied species. This indicates a fundamental reorganization of the food web structure in which resources that were once centered on benthic prey and detritus are now increasingly supplemented or replaced by planktonic food chains. This pattern indicates that eutrophication and habitat alteration are expanding pelagic food resources while reducing benthic niches. Consistent with this, we observed a decline in species with bottom-feeding ecology and a rise in species feeding on plankton or exhibiting omnivory (especially in YJ and YuJ). In trophic terms, the relative increase in mid-level consumers (as well as a decrease in top predators) indicates that the food webs are becoming shorter or more truncated, possibly due to the loss of large predators and an increase in the abundance of medium-sized generalists. At the size level, smaller fishes are favored, likely reflecting selective pressures for early maturity and fast growth under stressful, resource-variable conditions. These trait shifts imply a reduced set of functional roles within the ecosystem.

Functionally, the lower FRic in 2023 indicates that the community occupies a narrower trait space. This loss of available niche space is a classic signature of ecosystem degradation, wherein specialist species with unique trait combinations are filtered out. The concurrent increase in functional evenness (FEve) indicates that the remaining species occupy this reduced functional space more uniformly, with no single trait group dominating, which is characteristic of functional homogenization. Indeed, ecologists have noted that stressed ecosystems often exhibit functional homogenization as tolerant generalists replace a diverse array of specialists [34]. Similarly, increased functional dispersion and specialization suggest that while overall diversity has declined, a few species with novel or extreme traits (extremophiles of eutrophic conditions) have emerged. In summary, these trait-based changes from benthivores to planktivores, from large- to small-bodied species, and from diverse to compressed niches point to a simplification of ecological functions. Theoretically, this loss of functional richness will weaken ecosystem stability and resilience. Our results mirror those reported for other degraded ecosystems; for example, trait-space contraction has been documented in Caribbean reef fish assemblages following habitat loss [34]. Collectively, our functional analyses indicate that the Yujiang and Guijiang river ecosystems have undergone substantial functional reassembly, which may reduce their capacity to buffer against future environmental disturbances.

Overall, the trends in functional diversity are mixed but troubling. In subtropical rivers, urban segments have been reported to harbor higher species richness but lower functional richness compared with natural reaches, reflecting an influx of generalists that do not expand the trait space [27]. In Texas, a study found that functional evenness and dispersion actually increased in the more urban assemblage due to colonization by a few generalist invaders (tilapia and tolerant sunfish) after the extirpation of local specialists [1]. Such paradoxes indicate that the rises in diversity indices may mask biotic homogenization. Meta-analyses corroborate that freshwater communities worldwide are increasingly dominated by warm-water, disturbance-tolerant species. For example, a study showed a global shift toward warm- and slow-water-adapted fishes over recent decades; this “thermophilization” was strongest in rivers already modified by humans [4]. Likewise, another study documented pervasive fish biodiversity changes in >50% of rivers globally, with biotic communities becoming more similar across regions (homogenization) and only 14% of rivers remaining relatively intact [40]. Thus, although local species richness may not always decline, the character of fish assemblages is changing decisively, a shift that is captured by functional metrics even when taxonomic metrics remain unchanged.

Shifts in fish trait composition have important ecosystem consequences. The loss of functionally unique specialists (e.g., large predators, cold-water obligates) and the gain of redundant generalists alter the food-web dynamics, nutrient cycling, and ecosystem resilience [41]. For instance, if the numbers of large piscivores decline and small omnivores proliferate, the altered predation regime may have cascading effects and change invertebrate and plant communities. Previous research emphasizes that biodiversity loss involves not only reductions in species numbers but also shifts in trophic roles and degrees of specialization, wherein some ecosystem processes may be disproportionately degraded [27]. In this sense, even modest taxonomic changes can hide major functional shifts. Eastwood et al. (2023) illustrated this using sedimentary DNA, finding that pollutant-driven losses of microbial taxa changed the prevalence of key functional genes (nitrogen metabolism and degradation pathways), which suggested that fundamental ecosystem functions were disrupted by community reorganization [41]. By analogy, in fish assemblages, we expect that trait shifts (e.g., toward a smaller body size or more tolerant physiology) will modify the rates of nutrient flux, organic matter breakdown, and food web connectance.

Our decadal surveys revealed declines in taxonomic diversity and functional richness. However, the simultaneous increases in FEve and FDis indicated that the functional response was not a simple homogenization process. Instead, the remaining assemblages occupied a smaller but more evenly structured trait space, with some divergent trait positions. This pattern suggests trait-space compression with functional divergence, likely due to the decline in sensitive or intermediate trait combinations and the persistence of tolerant generalists and a few functionally distinct species [36]. At the broader community level, this pattern is consistent with the hypothesis that declines in specialist species can promote biotic homogenization and functional simplification [42]. Similarly, intensive land-use change has been shown to favor warm-water, slow-flow species at the expense of stream-adapted endemic species. Our results mirror these trends: benthic and large-bodied species were replaced by small, tolerant fishes, including tilapias, resulting in a contracted functional niche space [4]. Other subtropical case studies agree with our results: highly urbanized watersheds exhibit substantially lower native and functional fish diversity than forested systems. Overall, the loss of specialists and the proliferation of generalists reduce functional complementarity, making ecosystems less resilient. Theoretically, this simplification of traits can impair ecosystem functioning and stability, eroding the buffering capacity of communities against disturbance [42].

Changes in functional traits may also impair ecosystem services. Freshwater fishes contribute to services such as water purification through trophic control of algae, fisheries production, and even flood mitigation through bioturbation and regulation of aquatic vegetation. A community dominated by tolerant, generalist feeders may provide fewer of these services than one rich in specialists. For example, replacement of insectivorous stream fishes by more omnivorous or detritivorous species may alter leaf-litter breakdown rates and nutrient retention [27]. Moreover, functional redundancy theory predicts that the loss of unique traits reduces ecosystem resilience because communities with a lower trait diversity have fewer insurance species to buffer against novel stressors. Thus, the consistent patterns of functional homogenization and loss seen in our study suggest that ecosystem stability and service provision could be compromised in the long run. In sum, trait-based declines flag not only biodiversity loss but also potential erosion of ecosystem health and function.

### 4.3. Water Quality and Land-Use Change as Dominant Drivers of Fish Community Reorganization

Multivariate analyses highlighted water-quality and land-cover variables as key predictors of community change. In particular, dissolved oxygen, temperature, and percent impervious cover emerged as top drivers in the RDA and random forest models. This echoes findings from other studies in intensively managed rivers. In the Han River (China), a previous study applying a random forest model showed that hydrological factors (water level and width) best explained α-diversity, whereas water quality factors (DO, nutrients, and chlorophyll-a) dominated the β-diversity patterns [2]. In our case, increased nutrients and reduced DO likely promoted taxonomic turnover and trait shifts. Environmental filtering thus appears more important than spatial dispersal in structuring these fish assemblages. Climatic and anthropogenic factors, including precipitation, temperature, the presence of invasive species, and human population density, have also been identified as key drivers of temporal variation in fish functional diversity [5]. The strong correlations between diversity loss and eutrophication in our study suggest that simple water quality proxies can effectively signal biotic integrity. Indeed, trait-based indices have been shown to respond predictably to chemical stress [35]. Overall, our models demonstrate that oxygen availability and nutrient load are important predictors associated with fish community assembly, in line with the predictions of trait environment theory [6].

The observed trends have urgent implications for biodiversity conservation. Rapid urban and agricultural expansion is associated with reduced fish functional integrity in these rivers [43]. Conservation strategies should therefore prioritize mitigating these stressors. Protecting and restoring riparian forests would reduce runoff and maintain habitat heterogeneity [20]. Likewise, controlling nutrient inputs (e.g., through wastewater treatment or buffer zones) would help prevent eutrophication, which favors tolerant invaders [36]. Importantly, trait-based metrics emerged in our study as sensitive indicators of ecosystem degradation. Our findings support the use of functional richness and trait-convergence indices in biomonitoring programs [1]. Management targets should aim to conserve not only species richness but also functional diversity, for example, by protecting habitats that support specialist species. In rapidly urbanizing rivers, green infrastructure and connected floodplains can buffer streams against extreme flows and pollution, thereby helping to maintain fish diversity [35]. Overall, our results underscore that safeguarding freshwater biodiversity in subtropical rivers demands integrated land–water management and continuous monitoring of functional metrics [6].

Finally, our environmental predictor analysis integrated these patterns and indicated that deteriorating water quality and urban land use were strongly associated with fish community reorganization. The shift from geographic factors as dominant drivers in 2013 to water quality and land use in 2023 underscores how local environmental stress is increasingly overriding regional biogeographic constraints. The RDA and random forest models identified dissolved oxygen and temperature as the most important variables, followed by land-cover metrics, including bare land, cropland, and impervious surface. This pattern is consistent with the urban stream syndrome concept, which proposes that urbanization, through increasing impervious cover and channelization, fundamentally alters stream habitats, reducing connectivity and biodiversity [32]. Our findings are also consistent with previous studies showing that local anthropogenic disturbances, such as land-use change and hydrological alteration, can have stronger effects on fish diversity than broader climatic factors [44]. In our study, chlorophyll-a was negatively correlated with diversity, whereas dissolved oxygen was positively correlated, highlighting nutrient enrichment and hypoxia as major stressors affecting fish communities. Overall, we found that an intensified human footprint, reflected in urban and agricultural land use, has increased nutrient inputs and altered stream habitats in ways that drive the predictable loss of sensitive species and functional guilds. This conclusion is consistent with other studies. From temperate streams in North America to tropical rivers in Brazil, land-use change promotes eutrophication and habitat simplification, which in turn leads to simplified fish assemblages. Instead of climatic or large-scale factors, it is these local catchment changes that are the current main determinants of fish community composition [43]. Our multi-model results reinforce the importance of water quality (especially DO) and land-cover integrity for maintaining fish biodiversity.

In summary, this study’s decadal analysis provides strong evidence of land-use-driven reorganization in river fish assemblages. To improve river management, we advocate coordinated, long-term monitoring that combines taxonomic surveys with trait and ecosystem function indicators. This will require cross-river collaborations, consistent protocols, and inclusion of novel data sources (eDNA, remote sensing, etc.). In practice, managers should focus on preserving the remaining natural habitats and connectivity, controlling pollution and invasive species, and explicitly tracking changes in functional trait diversity, not just species counts [31]. The combination of a lower FRic but higher FEve and FDis should be interpreted as indicative of trait-space contraction accompanied by functional divergence among the remaining species, rather than as a simple increase or decrease in functional diversity.

A limitation of this study is that the 2013 dataset was obtained from a historical survey, whereas the 2023 dataset was collected through our resurvey. Although both surveys were restricted to comparable spring periods and the same fixed sites, differences in sampling protocols, field conditions, or historical recording procedures may have affected direct abundance comparisons. Therefore, abundance changes should be interpreted cautiously. To reduce taxonomic bias, species nomenclature was standardized across years using FishBase and regional monographs before analysis. Despite these limitations, the consistent changes in taxonomic composition, functional traits, and environmental correlates support the interpretation of decadal reorganization of fish assemblages.

## 5. Conclusions

Our results show that in the past decade, land-use change and water quality degradation have promoted the significant reorganization of river fish communities. By combining a decadal dataset with a trait-based framework, this study highlights the value of functional indicators for detecting fish community reorganization in subtropical rivers beyond changes in species richness alone. We observed shifts toward tolerant, generalist species and declines in functional uniqueness, even where taxonomic richness did not decrease. These trends mirror global patterns of freshwater biotic changes, with more than half of the world’s rivers showing marked community shifts (often toward homogenization). For conservation, our findings highlight the need to conserve trait diversity and ecosystem functions, not merely species counts. We recommend that biomonitoring programs adopt trait-based metrics to detect early warning signs of degradation, and that management plans prioritize maintaining intact watershed forests, riparian buffers, and connectivity. Only by integrating taxonomic, functional, and ecosystem perspectives can we hope to safeguard riverine biodiversity and the services it provides in an era of accelerating environmental changes.

## Data Availability

The raw data supporting the results of this study are available in the Supplementary Data.

## Supplementary Materials

The following supporting information can be downloaded at: https://www.mdpi.com/article/10.3390/ani16142253/s1. Table S1: Geographic coordinates of the sampling sites in Guangxi rivers, China; Table S2: Functional trait categories, coding scheme, and data sources used for fish trait assignment; Table S3: Functional traits of fish species recorded in Guangxi rivers in 2013; Table S4: Functional traits of fish species recorded in Guangxi rivers in 2023; Table S5: Effect sizes of environmental factors (one-way ANOVA); Table S6: Effect sizes of community diversity metrics; Table S7: Effect sizes of functional diversity metrics; Figure S1: Validation diagnostics of the Random Forest model; Figure S2: Partitioning of fish community β-diversity into turnover and nestedness-resultant components between 2013 and 2023; Supplementary Methods: Detailed formulas used to calculate species richness, the Shannon–Wiener diversity index, and Pielou’s evenness index; Supplementary Data: Raw datasets used in this study, including fish abundance, water-quality variables, land-use variables, and sampling-site information.
