# Supplementary material for: Decadal Land-Use Change and Water Quality Degradation Reshape the Functional Structure of Fish Assemblages in Guangxi Rivers, China
Source: Animals (Basel). 2026 Jul 21;16(14):2253. doi: 10.3390/ani16142253 (PMC13403959; doi:10.3390/ani16142253)
Supplement: Supplementary file 1 [file animals-16-02253-s001.zip › Supplementary materals.pdf]

Table S1. Geographic coordinates of the sampling sites in Guangxi rivers, China.

Site	Longitude	Latitude
GJ1	110.6751	25.6137
GJ2	110.4999	24.77099
GJ3	110.3745	25.59842
GJ4	110.8098	24.16213
GJ5	110.7109	24.58386
GJ6	110.8214	24.82923
YJ1	105.0558	24.48806
YJ2	105.9649	24.16554
YJ3	105.9735	24.18621
YJ4	106.4012	24.13015
YJ5	106.6616	23.78184
YJ6	107.1082	23.5867
YJ7	107.575	23.30631
YJ8	107.6417	23.27056
YuJ1	110.0659	23.34294
YuJ2	109.5891	22.95578
YuJ3	108.8751	22.87994
ZY1	107.0741	22.1405
ZY2	106.8586	22.34593
ZY3	107.4034	22.49643
ZY4	107.9583	22.69846

Table S2. Functional trait categories, coding scheme, and data sources used for fish trait assignment.

Trait	Data type	Coding scheme	Main source
Body Shape	Categorical	fusiform/normal; elongated; compressed; short and/or deep-bodied; eel-like	FishBase; regional monographs
Demers Pelag	Categorical	demersal; benthopelagic; pelagic/pelagic-neritic	FishBase "DemersPelag"
Feeding Path	Categorical	benthic; pelagic; omnivore	FishBase trophic ecology/diet information
Troph	Continuous	numerical trophic level	FishBase "Troph"
Max Length TL	Continuous	maximum total length, cm	FishBase maximum length record

Table S3. Functional traits of fish species recorded in Guangxi rivers in 2013.

Species	Body Shape	Demers Pelag	Feeding Path	Troph	Max Length TL
Siniperca liuzhouensis	fusiform / normal	benthopelagic	benthic	3.96000004	39.4799995
Parabramis pekinensis	short and / or deep	benthopelagic	benthic	2	55
Cirrhinus mrigala	elongated	demersal	benthic	2.26999998	99
Spinibarbus hollandi	elongated	benthopelagic	benthic	3.22000003	34
Discogobio tetrabarbatus	elongated	benthopelagic	benthic	2.3599999	16.5
Garra orientalis	elongated	benthopelagic	benthic	2	20
Gobiobotia kolleri	elongated	benthopelagic	benthic	3.25999999	15.25
Hemibarbus maculatus	fusiform / normal	benthopelagic	benthic	3.45000005	47
Mesocyprinus longzhouensis	compressed	benthopelagic	omnivore	2.8	20
Pseudohemiculter dispar	elongated	benthopelagic	benthic	3.20000005	29.1000004
Sarcocheilichthys parvus	fusiform / normal	benthopelagic	benthic	3.21000004	10.6099997
Mylopharyngodon piceus	fusiform / normal	demersal	benthic	3.19000006	180
Distoechodon tumirostris	compressed	benthopelagic	omnivore	2.7	31.84000015
Acheilognathus barbatulus	fusiform / normal	benthopelagic	benthic	2.23000002	8.69999981
Chanodichthys mongolicus	fusiform / normal	benthopelagic	pelagic	3.29999995	122
Culter recurviceps	elongated	benthopelagic	benthic	3.24000001	54.5999985
Hemibagrus macropterus	elongated	demersal	benthic	3.57999992	54.2000008
Clarias gariepinus	elongated	benthopelagic	benthic	3.75999999	170
Beaufortia kweichowensis	elongated	demersal	benthic	2.69000006	8
Coilia grayii	elongated	pelagic-neritic	pelagic	3.44000006	40.2599983
Psetulohemiculter hainanensis	compressed	benthopelagic	omnivore	3.2	24
Vanmanenia pingchowensis	elongated	demersal	benthic	2.55999994	13.8999996

Acheilognathus tonkinensis	fusiform / normal	benthopelagic	benthic	2.05999994	12.1999998
Pseudogobio guilinensis	elongated	benthopelagic	benthic	3.28999996	20
Hypophthalmichthys nobilis	fusiform / normal	benthopelagic	benthic	2.82999992	178.119995
Culter alburnus	elongated	benthopelagic	benthic	3.28999996	103.010002
Hypophthalmichthys molitrix	fusiform / normal	benthopelagic	pelagic	2.11999989	120
Ctenopharyngodon idella	fusiform / normal	benthopelagic	benthic	2	150
Abbottina rivularis	fusiform / normal	benthopelagic	benthic	3.28999996	18.8999996
Pseudorasbora parva	fusiform / normal	benthopelagic	pelagic	3.05999994	12.5
Sinibotia robusta	elongated	demersal	benthic	3.21000004	18
Hemibarbus labeo	fusiform / normal	benthopelagic	benthic	3.45000005	67
Channa maculata	elongated	benthopelagic	benthic	3.66000009	20
Ptychidio jordani	elongated	demersal	benthic	2.11999989	37
Rhodeus ocellatus	fusiform / normal	benthopelagic	benthic	2.51999998	11.2200003
Clarias fuscus	elongated	demersal	benthic	3.74000001	29.8899994
Schistura fasciolata	elongated	benthopelagic	benthic	2.98000002	12
Sinibotia pulchra	elongated	demersal	benthic	3.34999991	10
Cobitis sinensis	elongated	benthopelagic	benthic	3.27999997	15.3000002
Monopterus albus	eel-like	demersal	benthic	2.88000011	122
Squaliobarbus curriculus	elongated	benthopelagic	benthic	3.23000002	48.7999992
Macrognathus aculeatus	eel-like	benthopelagic	benthic	3.30999994	38
Sinibrama macrops	fusiform / normal	benthopelagic	benthic	3.18000007	22.7000008
Siniperca knerii	fusiform / normal	benthopelagic	benthic	3.92000008	35.5
Hemibagrus guttatus	fusiform / normal	demersal	benthic	3.66000009	89.1800003
Hemibarbus medius	elongated	benthopelagic	benthic	3.32999992	26.2000008
Mastacembelus armatus	elongated	demersal	benthic	2.77999997	90

Glyptothorax fokiensis fokiensis	elongated	benthopelagic	benthic	3.2	12.5
Opsariichthys bidens	fusiform / normal	benthopelagic	pelagic	3.19000006	26.1000004
Silurus asotus	elongated	demersal	pelagic	4.42999983	130
Siniperca scherzeri	fusiform / normal	benthopelagic	benthic	3.94000006	37.4000015
Squalidus wolterstorffi	elongated	benthopelagic	benthic	3.25999999	15.8000002
Cirrhinus molitorella	fusiform / normal	benthopelagic	benthic	2	55
Squalidus argentatus	elongated	benthopelagic	benthic	3.30999994	22.9400005
Coreoperca whiteheadi	fusiform / normal	benthopelagic	pelagic	4.05999994	26.6000004
Pseudobagrus crassilabris	elongated	demersal	benthic	3.51999998	39.6500015
Traccatichthys pulcher	elongated	benthopelagic	benthic	2.72000003	12
Osteochilus salsburyi	fusiform / normal	benthopelagic	benthic	2.11999989	20.1000004
Schistura incerta	elongated	demersal	benthic	2.98000002	14.3000002
Pseudobagrus vachellii	elongated	demersal	benthic	3.52999997	42.2999992
Acrossocheilus kreyenbergii	elongated	benthopelagic	benthic	2.74000001	18.8500004
Zacco platypus	fusiform / normal	benthopelagic	benthic	3.04999995	22.5
Cyprinus carpio	fusiform / normal	benthopelagic	benthic	3.38000011	120
Oreochromis mossambicus	fusiform / normal	benthopelagic	pelagic	2.17000008	47.5800018
Odontobutis sinensis	elongated	demersal	benthic	3.23000002	18.1000004
Rhinogobius giurinus	fusiform / normal	demersal	benthic	3.21000004	12.1000004
Carassius auratus	fusiform / normal	benthopelagic	benthic	2.77999997	48
Tachysurus fulvidraco	elongated	demersal	benthic	3.5	34.5
Misgurnus anguillicaudatus	elongated	demersal	benthic	3.24000001	34.1599999
Oreochromis niloticus	fusiform / normal	benthopelagic	benthic	2.02999997	73.199997
Toxabramis houdemeri	elongated	benthopelagic	benthic	3.16000009	18.0599995
Hemiculter leucisculus	fusiform / normal	benthopelagic	benthic	2.75	29

Table S4. Functional traits of fish species recorded in Guangxi rivers in 2023.

Species	Body Shape	Demers Pelag	Feeding Path	Troph	Max Length TL
Squalidus atromaculatus	elongated	benthopelagic	benthic	3.220000029	11
Ictalurus punctatus	fusiform / normal	demersal	benthic	4.159999847	132
Siniperca scherzeri	fusiform / normal	benthopelagic	benthic	3.940000057	37.40000153
Hemibagrus guttatus	fusiform / normal	demersal	benthic	3.660000086	89.18000031
Channa maculata	elongated	benthopelagic	benthic	3.660000086	20
Beaufortia pingi	elongated	demersal	benthic	2.75	6.099999905
Siniperca undulata	fusiform / normal	benthopelagic	benthic	3.650000095	15
Hemiculter leucisculus	fusiform / normal	benthopelagic	benthic	2.75	29
Ctenopharyngodon idella	fusiform / normal	benthopelagic	benthic	2	150
Squaliobarbus curriculus	elongated	benthopelagic	benthic	3.230000019	48.79999924
Hemibarbus labeo	fusiform / normal	benthopelagic	benthic	3.450000048	67
Macrognathus aculeatus	eel-like	benthopelagic	benthic	3.309999943	38
Pseudobagrus crassilabris	elongated	demersal	benthic	3.519999981	39.65000153
Hemibagrus macropterus	elongated	demersal	benthic	3.579999924	54.20000076
Siniperca knerii	fusiform / normal	benthopelagic	benthic	3.920000076	35.5
Sinibrama macrops	fusiform / normal	benthopelagic	benthic	3.180000067	22.70000076
Ancherythroculter lini	elongated	benthopelagic	benthic	3.200000048	31.47999954
Ptychidio macrops	elongated	benthopelagic	benthic	2.24000001	24.52000046
Acrossocheilus kreyenbergii	elongated	benthopelagic	benthic	2.74000001	18.85000038
Spinibarbus denticulatus	elongated	benthopelagic	benthic	3.25	50.63000107
Squalidus wolterstorffi	elongated	benthopelagic	benthic	3.25999999	15.80000019
Microphysogobio tungtingensis	elongated	benthopelagic	benthic	3.25	14.02999973
Glyptothorax fokiensis fokiensis	elongated	demersal	benthic	3.2	12.5

Microphysogobio fukiensis	elongated	benthopelagic	benthic	3.220000029	11.19999981
Tilapia galilaea	compressed	benthopelagic	omnivore	2.3	41
Rhodeus ocellatus	fusiform / normal	benthopelagic	benthic	2.519999981	11.22000027
Spinibarbus hollandi	elongated	benthopelagic	benthic	3.220000029	34
Beaufortia kweichowensis	elongated	demersal	benthic	2.690000057	8
Pseudogobio guilinensis	elongated	benthopelagic	benthic	3.289999962	20
Culter recurviceps	elongated	benthopelagic	benthic	3.24000001	54.59999847
Psetulohemiculter hainanensis	compressed	benthopelagic	omnivore	3.2	24
Toxabramis houdemeri	elongated	benthopelagic	benthic	3.160000086	18.05999947
Cyprinus carpio var. Quanzhounensis	fusiform / normal	benthopelagic	benthic	3.380000114	120
Schistura fasciolata	elongated	benthopelagic	benthic	2.980000019	12
Hemibarbus maculatus	fusiform / normal	benthopelagic	benthic	3.450000048	47
Hemibarbus umbrifer	elongated	benthopelagic	benthic	3.289999962	19.5
Tachysurus fulvidraco	elongated	demersal	benthic	3.5	34.5
Carassius auratus	fusiform / normal	benthopelagic	benthic	2.779999971	48
Hemibarbus medius	elongated	benthopelagic	benthic	3.329999924	26.20000076
Channa gachua	elongated	benthopelagic	pelagic	3.829999924	32.90000153
Zacco platypus	fusiform / normal	benthopelagic	benthic	3.049999952	22.5
Parabotia lijiangensis	elongated	demersal	benthic	3.299999952	19.25
Rhinogobius leavelli	fusiform / normal	benthopelagic	benthic	3.329999924	10.73999977
Cyprinus carpio	fusiform / normal	benthopelagic	benthic	3.380000114	120
Hypophthalmichthys molitrix	fusiform / normal	benthopelagic	pelagic	2.119999886	120
Cirrhinus molitorella	fusiform / normal	benthopelagic	benthic	2	55
Labeo rohita	fusiform / normal	benthopelagic	pelagic	2.190000057	200
Opsariichthys bidens	fusiform / normal	benthopelagic	pelagic	3.190000057	26.10000038

Parachromis managuensis	fusiform / normal	benthopelagic	pelagic	3.960000038	55
Cirrhinus mrigala	elongated	demersal	benthic	2.269999981	99
Pseudorasbora parva	fusiform / normal	benthopelagic	pelagic	3.059999943	12.5
Onyclwstoma gerlachi	fusiform	benthopelagic	omnivore	2.7	31.6
Pseudohemiculter dispar	elongated	benthopelagic	benthic	3.200000048	29.10000038
Gobiobotia meridionalis	elongated	benthopelagic	benthic	3.24000001	13.30000019
Oreochromis niloticus	fusiform / normal	benthopelagic	benthic	2.029999971	73.19999695
Silurus asotus	elongated	demersal	pelagic	4.429999828	130
Coilia grayii	elongated	pelagic-neritic	pelagic	3.440000057	40.25999832
Tilapia zillii	compressed	benthopelagic	omnivore	2.4	40
Culter alburnus	elongated	benthopelagic	benthic	3.289999962	103.0100021
Mylopharyngodon piceus	fusiform / normal	demersal	benthic	3.190000057	180
Liniparhomaloptera qiongzhongensis	elongated	demersal	benthic	2.680000067	8.100000381
Megalobrama terminalis	fusiform / normal	benthopelagic	benthic	3.25	60
Saurogobio dabryi	elongated	benthopelagic	benthic	3.329999924	27.29999924
Microphysogobio labeoides	elongated	benthopelagic	benthic	3.220000029	11.22000027
Discogobio tetrabarbatus	elongated	benthopelagic	benthic	2.359999895	16.5
Megalobrama amblycephala	fusiform / normal	benthopelagic	benthic	3.339999914	200
Pelteobagrus vachelli	elongated	demersal	omnivore	3.7	42.3
Osteocheilus salsburyi	fusiform/ normal	benthopelagic	omnivore	2.11999989	20.1000004
Rhinogobius duospilus	fusiform / normal	demersal	benthic	3.25999999	7.559999943
Plagiognathops microlepis	fusiform / normal	benthopelagic	benthic	3.25999999	70
Pseudobagrus pratti	elongated	benthopelagic	benthic	3.529999971	40.02000046
Sarcocheilichthys parvus	fusiform / normal	benthopelagic	benthic	3.210000038	10.60999966
Rhinogobius duospilus	fusiform / normal	demersal	benthic	3.25999999	7.559999943

Carassioides acuminatus	fusiform / normal	benthopelagic	benthic	2.900000095	25.43000031
Pterygoplichthys disjunctivus	elongated	demersal	benthic	2	70
Squalidus argentatus	elongated	benthopelagic	benthic	3.309999943	22.94000053
Xenocypris macrolepis	fusiform / normal	benthopelagic	benthic	3.220000029	42.20999908
Hypophthalmichthys nobilis	fusiform / normal	benthopelagic	benthic	2.829999924	178.1199951
Distoechodon tumirostris	fusiform / normal	benthopelagic	benthic	3.200000048	31.84000015
Silurus cochinchinensis	elongated	demersal	carnivore	3.8	40
Acheilognathus tonkinensis	fusiform / normal	benthopelagic	benthic	2.059999943	12.19999981
Oxyeleotris marmorata	fusiform / normal	demersal	benthic	3.900000095	79.30000305
Acrossocheilus longipinnis	elongated	benthopelagic	benthic	2.730000019	21.12999916
Spinibarbus sinensis	elongated	benthopelagic	benthic	2.630000114	61
Odontobutis sinensis	elongated	demersal	benthic	3.230000019	18.10000038
Coreoperca whiteheadi	fusiform / normal	benthopelagic	pelagic	4.059999943	26.60000038
Pelteobagrus intermedius	elongated	demersal	benthic	3.410000086	21.11000061
Rhinogobius giurinus	fusiform / normal	demersal	benthic	3.210000038	12.10000038

Table S5. Effect sizes of environmental factors (one-way ANOVA)

Variable	ANOVA	η^2	Cohen's d	Effect size	Trend
Temperature (°C)	F = 0.14, p = 0.7134	0.003	-0.11	Small	No change
SpCond (μs/c)	F = 0.79, p = 0.3784	0.019	-0.27	Medium	Decrease
pH	F = 0.07, p = 0.7933	0.002	-0.08	Small	No change
Chlorophyll.a(μg/L)	F = 6.17, p = 0.0172	0.134	0.77	Large	Increase
DO (mg/L)	F = 6.17, p = 0.0173	0.134	-0.77	Large	Decrease

Note: η^2 = eta-squared effect size; Effect size = effect size classification based on Cohen's d (Small/Medium/Large/Very Large); Trend indicates the direction of changes from 2013 to 2023.

Table S6. Effect sizes of community diversity metrics

Diversity index	ANOVA	η^2	Cohen's d	Effect size	Trend
Beta diversity (Bray-Curtis)	F = 6.27, p = 0.001	0.136	1.78	Very Large	Increase
Shannon index	F = 10.74, p = 0.0022	0.212	-1.01	Very Large	Decrease
Species richness	F = 20.56, p = <0.001	0.339	-1.4	Very Large	Decrease
Pielou evenness	F = 0.75, p = 0.3926	0.018	-0.27	Medium	Decrease

Note: η^2 for Beta diversity was derived from PERMANOVA; η^2 for the remaining indices was derived from one-way ANOVA. Trend indicates the direction of change from 2013 to 2023.

Table S7. Effect sizes of functional diversity metrics

Functional diversity index	ANOVA	η^2	Cohen's d	Effect size	Trend
Species number	F = 20.56, p = <0.001	0.339	-1.4	Very Large	Decrease
Fric	F = 8.55, p = 0.0057	0.176	-0.9	Very Large	Decrease
Feve	F = 4.07, p = 0.0503	0.092	0.62	Large	Increase
Fdiv	F = 3.84, p = 0.0572	0.087	0.6	Large	Increase
Fdis	F = 2.23, p = 0.1432	0.053	0.46	Medium	Increase

Note: Species number = species count; Fric = functional richness; Feve = functional evenness; Fdiv = functional divergence; Fdis = functional dispersion. Trend indicates the direction of change from 2013 to 2023.

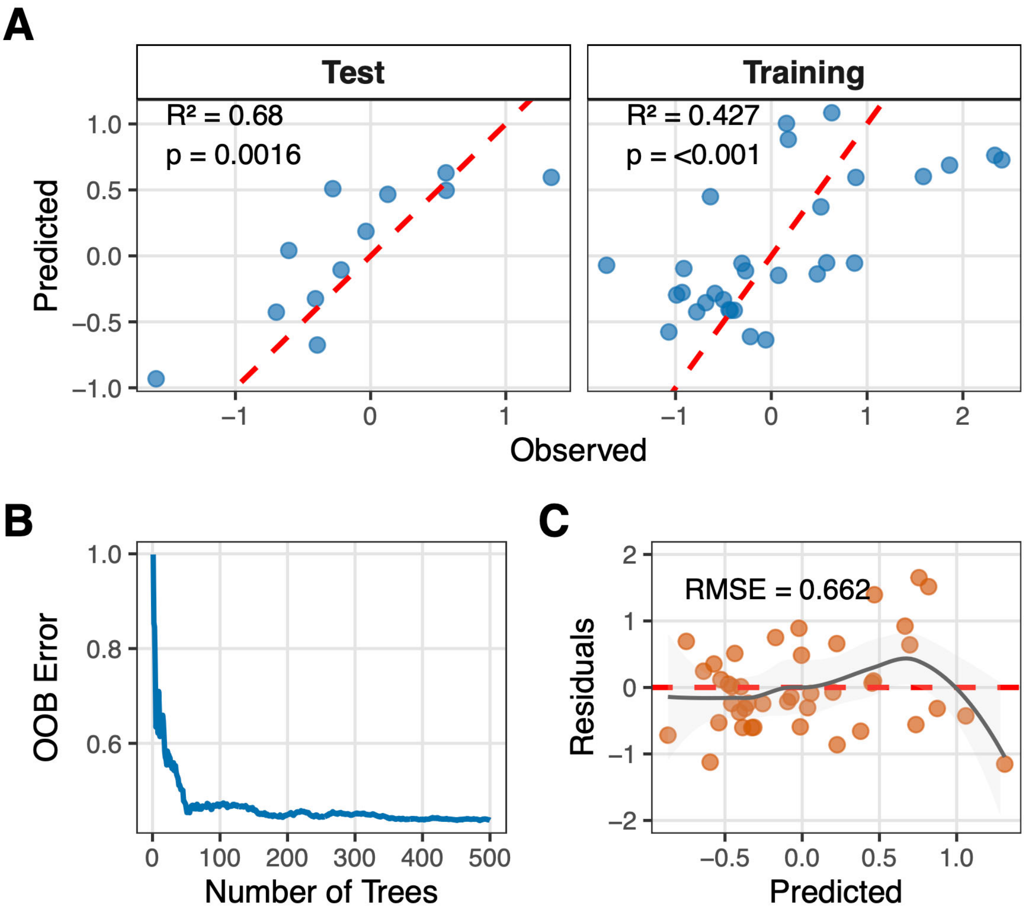

Figure S1. Validation diagnostics of the Random Forest model.

Note: Validation diagnostics of the Random Forest model. Model performance was evaluated using five-fold cross-validation, out-of-bag (OOB) error analysis, training–testing dataset comparison, and residual diagnostics. The validation results showed acceptable predictive performance and model stability, with an average cross-validation R^2 of 0.6307 ± 0.1976, RMSE of 0.6410 ± 0.1918, and MAE of 0.5296 ± 0.1651. OOB error stabilized after approximately 100 trees, and residuals were generally centered around zero.

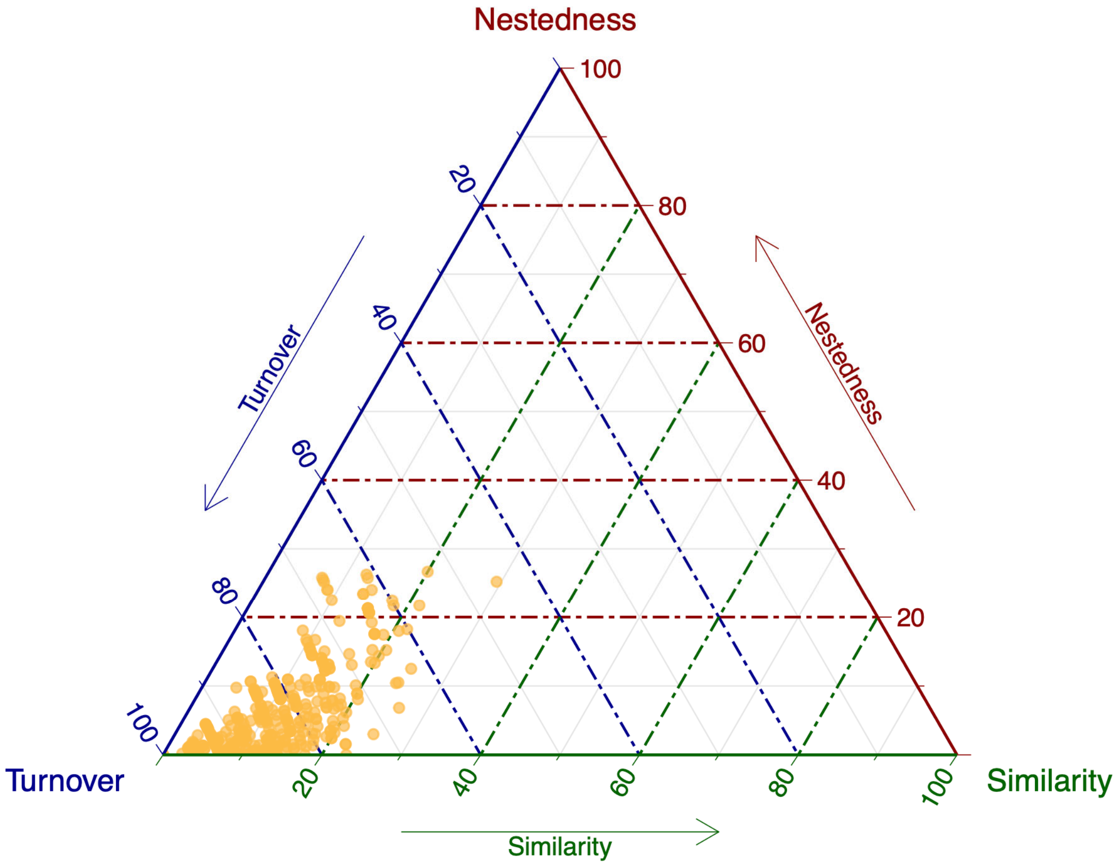

Figure S2. Partitioning of fish community β-diversity into turnover and nestedness-resultant components between 2013 and 2023.

Supplementary Methods

Species richness was calculated as the total number of species recorded in each fish assemblage:

$$S = \sum_{i=1}^{N} I(n_i > 0)$$

where S is species richness, N is the total number of species in the dataset,n_i is the abundance of species i, and $I(n_i > 0)$ is an indicator function equal to 1 when species i is present and 0 otherwise.

The Shannon–Wiener diversity index was calculated as:

$$H' = -\sum_{i=1}^{S} p_i \ln{(p_i)}$$

where H' is the Shannon–Wiener diversity index, S is species richness, and p_i is the relative abundance of species i, calculated as:

$$p_i = \frac{n_i}{N}$$

where n_i is the abundance of species i, and N is the total abundance of all species in the assemblage.

Pielou's evenness index was calculated as:

$$J = \frac{H'}{\ln{(S)}}$$

where J is Pielou's evenness index,$H'$is the Shannon–Wiener diversity index, and S is species richness. The value of J ranges from 0 to 1, with higher values indicating a more even distribution of individuals among species.
